# The future of graduate and postdoctoral training in the biosciences

**DOI:** 10.7554/eLife.32715

**Published:** 2017-10-19

**Authors:** Peter Hitchcock, Ambika Mathur, Jabbar Bennett, Patricia Cameron, Christine Chow, Philip Clifford, Robert Duvoisin, Andrew Feig, Kevin Finneran, Diane M Klotz, Richard McGee, Mary O'Riordan, Christine Pfund, Christopher Pickett, Nancy Schwartz, Nancy E Street, Elizabeth Watkins, Jonathan Wiest, David Engelke

**Affiliations:** 1 Rackham Graduate SchoolUniversity of MichiganAnn ArborUnited States; 2 Graduate SchoolWayne State UniversityDetroitUnited States; 3 PediatricsWayne State UniversityDetroitUnited States; 4 Department of MedicineNorthwestern University Feinberg School of MedicineChicagoUnited States; 5 Office of the ProvostNorthwestern UniversityChicagoUnited States; 6 Graduate SchoolAugusta UniversityAugustaUnited States; 7 Department of ChemistryWayne State UniversityDetroitUnited States; 8 College of Applied Health SciencesUniversity of Illinois ChicagoChicagoUnited States; 9 Department of Physiology and Pharmacology, School of MedicineOregon Health and Science UniversityPortlandUnited States; 10 National Academy of SciencesWashington, DCUnited States; 11 Office of Education, Training, and International ServicesSanford Burnham Prebys Medical Discovery InstituteSan DiegoUnited States; 12 Faculty Affairs OfficeNorthwestern University Feinberg School of MedicineChicagoUnited States; 13 Department of MicrobiologyUniversity of Michigan Medical SchoolChicagoUnited States; 14 Wisconsin Center for Education ResearchUniversity of Wisconsin-MadisonMadisonUnited States; 15 Institute for Clinical and Translational ResearchUniversity of Wisconsin-MadisonMadisonUnited States; 16 Rescuing Biomedical ResearchAAASWashington, DCUnited States; 17 Department of PediatricsUniversity of ChicagoChicagoUnited States; 18 Department of Biochemistry and Molecular BiologyUniversity of ChicagoChicagoUnited States; 19 Southwestern Graduate SchoolUniversity of Texas Southwestern Medical CenterDallasUnited States; 20 Department of MicrobiologyUniversity of Texas Southwestern Medical CenterDallasUnited States; 21 Graduate DivisionUniversity of California, San FranciscoSan FranciscoUnited States; 22 Center for Cancer TrainingNational Cancer InstituteWashingtion, DCUnited States; 23 Graduate SchoolUniversity of Colorado Denver | Anschutz Medical CampusDenverUnited States

**Keywords:** grad school, postdoc, FOBGAPT, mentorship, careers in science, workforce diversity

## Abstract

This article summarizes the outcomes of the second national conference on the Future of Bioscience Graduate and Postdoctoral Training. Five topics were addressed during the conference: diversity in leadership positions; mentoring; modernizing the curriculum; experiential learning; and the need for better data on trainees. The goal of the conference was to develop a consensus around these five topics and to recommend policies that can be implemented by academic and research institutions and federal funding agencies in the United States.

## Introduction

For over two decades scientists, policy-makers, funders and academic leaders in the US have discussed the changing nature of careers for bioscientists. Are we training too many PhDs? Are we preparing our students adequately for future careers? And what steps must be taken to address the persistent lack of diversity in the scientific workforce? These discussions have intensified in the last few years, with publications that call for change (National Institutes of Health, 2012; National Academies, 2014; Alberts et al., 2014; Daniels, 2015; Gibbs et al., 2014; Gibbs and Marsteller, 2016) and conferences convened to advance the conversations and identify effective solutions (McDowell et al., 2015; Kimble et al., 2015).

**Figure 1.**
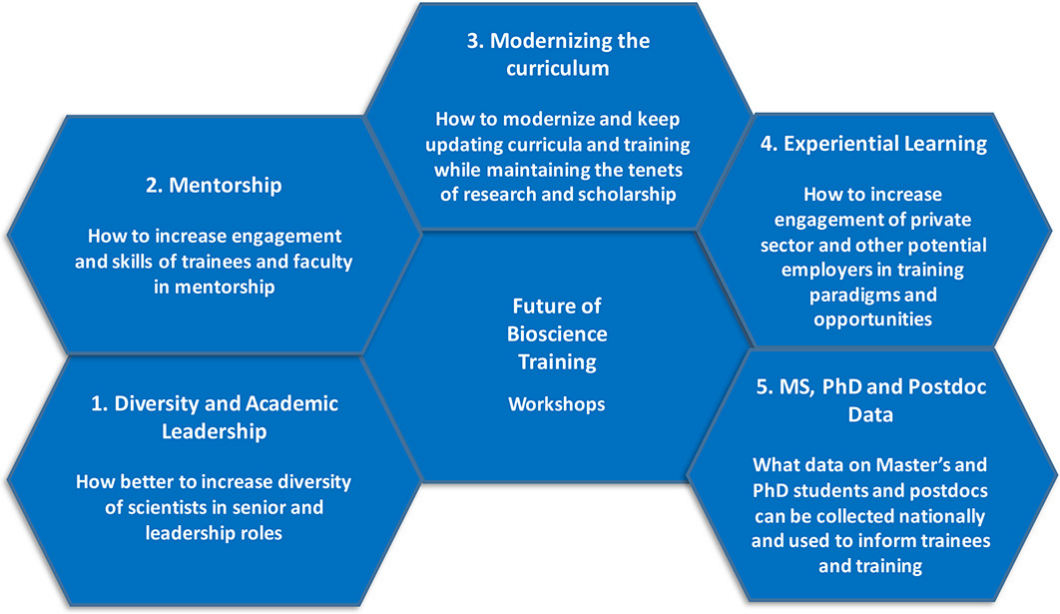
Working groups at the second national conference on the Future of Bioscience Graduate and Postdoctoral Training. Delegates at the conference discussed five different topics: diversity in leadership positions; mentoring; modernizing the curriculum; experiential learning; and the need for better data on trainees.

In 2015, the University of Michigan hosted the first Future of Bioscience Graduate and Postdoctoral Training Conference (FOBGAPT1). The goal of this conference was to discuss the previous proposals and to serve as the starting point for a second conference to develop policy recommendations. This article summarizes the discussions and recommendations from the second conference, FOBGAPT2, which was held in Denver in June 2017. The sections in the article correspond to the five workshops that ran throughout the two days of the conference (Figure 1). The five topics covered by the workshops had been identified by the organizing committee as those most in need of institutional and/or national policy solutions. Two important topics – the current stresses on federal funding in the US, and the mismatch between supply and demand for bioscientists in academia – were excluded, because they have been addressed previously (see, for example, Alberts et al., 2014; Levitt and Levitt, 2017). Nonetheless, the potential roles of federal funding agencies to impact policy changes were recognized and discussed, and specific recommendations to federal agencies were made.

**Figure 2.**
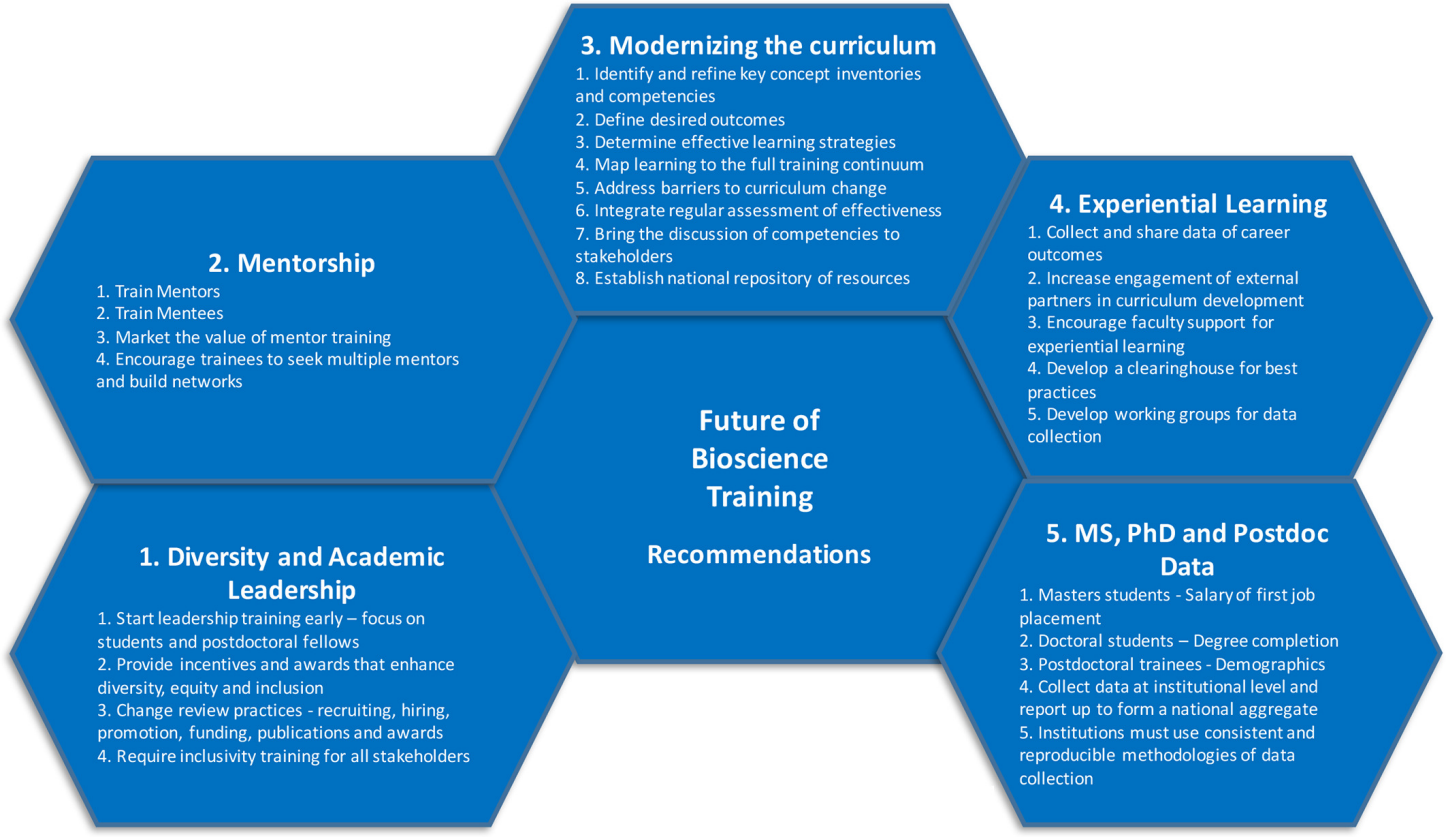
Recommendations from the second national conference on the Future of Bioscience Graduate and Postdoctoral Training. Each of the five working groups at the conference produced a list of recommendations aimed at academic and research institutions and funding agencies in the United States.

Each workshop was repeated five times over the course of two days and concluded with a session to refine recommendations. Attendees included university administrators and faculty, funding agency representatives, journal editors, postdoctoral fellows and graduate students. Each attendee was encouraged to participate in multiple workshops to help develop a broad consensus on recommendations. A longer version of this article, which includes a summary of the first conference, links to 'What Works' abstracts from conference participants and other resources, is available at the meeting website. The recommendations that emerged from the workshop are listed in Figure 2.

## Diversity and Academic Leadership: How to better increase the diversity of scientists in senior and leadership roles

Increasing the number of women and the number of scientists from underrepresented minorities in senior and leadership positions in the biosciences has been a challenge for decades (National Institutes of Health, 2012). Factors that impact a pathway toward successful leadership roles include awareness of and access to various career development resources, including mentors, and the presence of peers with similar experiences. Barriers to success include implicit biases that impact hiring, grant funding, peer review of publications, and recognition and awards. All too often, these barriers follow women and underrepresented scientists throughout their careers. Therefore, academic institutions, corporations, governmental and scientific organizations, funding agencies, publishers, and professional societies can play critical roles in promoting diversity among the ranks of senior leadership.

It is crucial to invest early in professional development and leadership training for graduate students and postdoctoral fellows. Scientists-in-training should be exposed to the variety of career paths available to them and encouraged to develop and communicate their professional and career goals to faculty and senior leaders. A climate of frank discussion between the trainee and research advisor regarding the trainee’s career interests must be fostered.

Institutional leaders must underscore the importance of diversity, and emphasize how an inclusive culture is essential for institutional success. It will be important to provide education and training to every member of an institution around these priorities. However, rather than framing this as mandatory training, a better strategy is to focus on specific topics and the need to comply with local and federal guidelines and expectations. Institutions should link efforts to create and sustain a more inclusive culture and climate to compensation and promotion.

Training in implicit bias is strongly encouraged for anyone involved in the review of applications for grants and fellowships and in decisions about jobs and promotion, and the use of blind review should be explored by universities and funding agencies. Once changes have been made, it is important to analyze outcomes and utilize these data and results to inform others (inside and outside academia) and encourage broader change.

Lastly, funding agencies should establish funding mechanisms to prepare postdoctoral fellows from underrepresented groups for transitions into faculty careers and leadership positions. For example, the National Institutes of Health (NIH) could create a new genre of individual career development awards for fellows from disadvantaged groups in the current 'K' kiosk of awards that already provide mentored training and protected time for advancement to faculty careers and/or leadership roles (such as division chief, chair and assistant/associate dean).

## Mentorship: How to Increase the Engagement and Skills of Trainees and Faculty in Mentorship

A growing literature provides strong evidence for the importance of mentoring, and a range of new approaches and resources have become available in the past decade (Pfund et al., 2016; McGee, 2016; National Academies, 2017; https://nrmnet.net/). However, too few mentors and their trainees have been introduced to them. An increased focus on mentoring will help us to: advance the skills of faculty in mentorship; improve the mentoring of trainees from diverse backgrounds; bring uniformity of excellence in mentoring across the range of institutions; and assess the effectiveness and impact of mentoring methodologies so that best practices can be identified and shared widely.

There are multiple dimensions to developing effective mentoring, and the quality of mentoring relationships is a responsibility shared by both mentors and mentees. Training provided to both groups should lead to insights into the dynamics that underlie a mentor-mentee relationship, sustain communication and allow each to be proactive in addressing issues that may arise. Mentees should build on mentoring experiences as they advance through their career and seek mentoring that is appropriate to the career stage (Lee et al., 2015). Mentees should build mentoring networks or mosaics, which can include formal and informal mentors, peer mentors and career mentors or coaches. Mentees should also be encouraged to seek mentors outside academia. Ideally, mentors should have the skills to provide career coaching to their mentees.

Institutions must effectively communicate to mentors and mentees the tangible positive impacts of building skills around mentoring: effective mentoring increases the productivity of the research group and improves mentor knowledge and satisfaction, which translates into changes in behavior that are evident to mentees (Pfund et al., 2014). Having a reputation as an effective mentor improves recruitment. When developing and advertising workshops, institutions should emphasize these positive attributes and departmental leaders should encourage faculty to participate.

Finally, there is a need for funding agencies to advocate for accountability for effective mentoring. This could be achieved by requesting an individual development plan, training and mentoring plans for all trainees on federal research or training grants, and annual reporting of mentoring activities in progress reports as a requirement for renewal of grant funding.

## Modernizing the Curriculum: How to Modernize (and keep updating) curricula and training while maintaining the tenets of research and scholarship

The remarkable breadth of career opportunities in the biosciences is driving the need for curriculum change. Graduate and postdoctoral researchers use their scientific training to make important contributions in academia, industry, government, health, communication, philanthropy, non-profits and outreach. These highly diverse modes of scholarship and employment require a broad array of skills, abilities and knowledge, but our training paradigms are still largely aimed at producing academic researchers.

Modernization, therefore, must involve aligning the learning objectives of the graduate curriculum with the wider spectrum of proficiencies that are valued in today’s workforce. A broad framework should be utilized to engage the scholarly community in a process that will intentionally and iteratively modernize curriculum and training programs. This framework should include skills in communication, teamwork and collaboration, leadership and development, and project management (see, for example, National Institute of General Medical Sciences, 2011; National Postdoctoral Association, 2017 for discussions of such skills and competencies). Training in these skills should be incorporated into the regular doctoral curriculum and should not be added on as optional or extra-curricular. The effectiveness of this training must also be assessed in the same manner as the regular curriculum (that is, with surveys of trainers and trainees).

Modernizing education and training in the biomedical sciences, without sacrificing the core values of research and scholarship, will be complex and challenging, and it will be important to involve faculty, trainees, alumni, professional societies and employers in evaluating the strengths and gaps in current programs. Work at individual institutions will be facilitated by establishing a national repository to disseminate and share relevant resources and best practices, such as that initiated by the American Society for Biochemistry and Molecular Biology (Fuhrmann, 2016).

## Experiential Learning: How to increase the engagement of private sector and other potential employers in training paradigms and opportunities

Engaging the private sector in biomedical education and training is critical in light of the fact that most biomedical PhD recipients will pursue careers outside of academia (National Institutes of Health, 2012). There is a need, therefore, to make education and training in the biosciences align with career paths. One approach is to create opportunities for experiential learning outside the research laboratory. For this to be successful, incentives and barriers to providing students and postdoctoral fellows with career-focused learning should be identified, and potential formats should be explored (such as internships and externships). For widespread adoption, practices from successful programs that can be implemented elsewhere should be shared. Since few industry representatives attended this conference, it is important to ensure that industrial employers are fully involved in future discussions.

Prior discussions of experiential programs for graduate students and postdocs have focused solely on the benefit to the student or postdoctoral fellow. It is apparent, however, that programs that have mutual benefit to the trainee, the university and the external partner are likely to be the most sustainable. Benefits for institutions include closer ties with employers, and greater awareness by graduate programs of the career opportunities outside academia. Benefits for employers include the ability to hire PhD scientists with a better match of soft skills. Benefits to the trainee include a deeper understanding of their career options and awareness of the non-academic work environment.

As graduate programs consider implementing experiential learning, there is a need to gather robust data on the impact of career-related experiences and programs. For example, will internships impact a student’s time to degree or a postdoc’s research productivity? The clearest data would be provided by a longitudinal analysis, relating internships to job satisfaction, career preparation and long-term success, but it is difficult to collect such data and to find suitable control groups. Several NIH-funded BEST programs have adopted internships and thus might provide natural study groups to follow as they enter the workforce (Mathur et al., 2015; Meyers et al., 20152016).

There are several challenges related to the uptake of experiential learning by training faculty and graduate programs. The major barrier among training faculty are concerns about time away from the laboratory and the potential for slowing research progress. Structures should be created within individual laboratories and graduate programs that are conducive both to continued productivity in the lab and career development/exploration. These activities should not require a commitment of more than one day per week, and some activities, such as volunteer consulting, could occur outside normal working hours (Schillebeeckx et al., 2013).

Launching an effective experiential learning or internship program can involve a significant amount of work, so it is important that effort is not duplicated. For example, while documents like non-disclosure agreements and memoranda of understanding will need to be customized, institutions would benefit from having standardized templates to use as starting points. Federal agencies that fund both training and research grants should provide specific guidance regarding the type and nature of experiential learning activities that are acceptable under the terms of their grants.

## MS, PhD and Postdoc Data: What data on Master’s and PhD students and postdocs can be collected nationally and used to inform trainees and training?

In 2014 the Council of Graduate Schools published a report, Understanding PhD Career Pathways for Program Improvement, that laid out a clear and persuasive rationale for tracking career outcomes: transparency for prospective students and postdocs; the improvement of program curricula; the development of institution-wide programming; and the development of faculty mentoring. There exists a widespread consensus on the need to collect, analyze and report career outcomes for Master’s students, PhD students and postdoctoral fellows and alumni at each career stage. Efforts to begin collecting data should address *what* information should be collected about trainees, *who* will be the audiences for this information, and *how* it will be used to influence curricula, mentoring, and other best practices.

Numerous schools have begun this effort, and various coalitions are working together to develop common and consistent methodologies for classifying and reporting job types within multiple sectors of the workforce. These initiatives will benefit all stakeholders. Institutions want access to data about their own trainees to drive curricular reform, develop co-programming, support local initiatives and for benchmarking. Prospective students and postdocs want clear information on a range of topics.

Master’s students are less well studied and less well understood than the rest of the biomedical workforce. For students enrolled in stand-alone Master's programs, or students who exit a PhD program and receive an Master's degree, the first job after graduation and starting salary are relevant. In particular such data will help prospective studies to assess the return on investment for stand-alone Master’s programs; such data will also reveal the merits and drawbacks of leaving a PhD program.

Data for PhD students should include time-to-degree and completion rates: these data should be broken out by program, URM/non-URM, gender, and citizenship. The data on completion rates needs to take account of those who withdraw from PhD programs with and without Master’s degrees.

Many institutions struggle to identify their postdoctoral populations, and national estimates are widely recognized to be unreliable (see, for example, National Academies, 2014). A collective effort to learn about who postdoctoral fellows are is the first step to providing them with the services they need to prepare for and enter meaningful careers. Demographic data should be available from institutional records and should not require surveying individual postdoctoral fellows or alumni.

Finally, national statistics should be aggregated from institutionally-collected data. In particular the National Science Foundation (NSF) should consider replacing the three national surveys it carries out (the Survey of Earned Doctorates, the Survey of Doctoral Recipients, and the Early Career Doctorate Survey) with surveys based on the aggregation of locally collected data as the latter would be more useful, more reliable and less expensive to collect. The funds that the NSF currently uses for national data collection could be redirected to institutions to help support their data collection efforts.

## Conclusion

At many levels, graduate education and postdoctoral training in the US are at a critical crossroads, and a wide range of stakeholders – academic and research institutions, funding agencies, learned and professional societies, and employers – must work together to shape how they will look in the future. Acting on the recommendations that emerged from the FOBGAPT2 conference in Denver (Figure 2) will, we are sure, lead to improvements in the training of young bioscientists for a wide range of careers.
